# How Restrictive Legislation Influences Antimicrobial Susceptibility in Selected Bacterial Isolates from the Canine Vagina

**DOI:** 10.3390/antibiotics13100946

**Published:** 2024-10-09

**Authors:** Anna Sophia Leps, Babette Klein, Marianne Schneider, Sandra Goericke-Pesch

**Affiliations:** 1 Unit for Reproductive Medicine—Clinic for Small Animals, University of Veterinary Medicine Hannover, 30559 Hannover, Germany; 2 Laboklin GmbH & Co. KG, 97688 Bad Kissingen, Germany

**Keywords:** antimicrobial resistance, antimicrobial susceptibility, veterinary microbiology, vaginal flora, antibiotics, canine reproduction, canine vaginal flora, dog

## Abstract

Antimicrobial stewardship is one of the cornerstones in the battle against antimicrobial resistance. Restrictive legislation aims to foster antimicrobial stewardship. Prophylactic prescription of antimicrobials is still a widespread practice in canine breeding management to prevent suspected infectious infertility. The aim of this study was to evaluate the efficacy of restrictive legislation in Germany (Veterinary Home Pharmacy Ordinance, TÄHAV) based on resistance profiles of common bacterial isolates from the vaginal tract by comparing the resistance situation before (time frame (TF1)) and after (TF2) its amendment. In total, results of 13,373 antimicrobial susceptibility tests of bacterial isolates of *Escherichia coli* (n = 5209), beta-hemolytic streptococci (n = 4010), and *Staphylococcus (Staph.) intermedius* group (n = 4154) derived from canine vaginal swabs were assessed. Antimicrobial susceptibility testing was performed on pure cultures using the broth microdilution method. Susceptibility to selected antimicrobials was evaluated. Susceptibility of *Escherichia coli* generally increased within TF2 with, however, a significant increase in the number of non-susceptible isolates to cefalexin (*p* < 0.0001). Beta-hemolytic streptococci exhibited good susceptibility to most antimicrobials. Susceptibility developed ambivalently within the *Staphylococcus intermedius* group. Despite an overall positive effect of increased susceptibility, an increase in non-susceptibility to single antimicrobials was detected, possibly indicating a need for refinement of the legislation.

## 1. Introduction

The development of penicillin in 1929 [1] marked the beginning of a revolution in the therapy of bacterial infections. Almost 100 years later, both human and veterinary medicine are facing a global crisis of antimicrobial resistance (AMR), with approximately 1.27 million deaths worldwide attributable to antimicrobial resistant infections in 2019 [2]. Antimicrobial resistance poses a major threat, with tremendous consequences for both public health and economic well-being [3]. It has been shown that also pet animals might act as a reservoir for antimicrobial resistance, and zoonotic transfer of resistance genes to humans is possible [4,5,6]. The risk of transmission is further promoted by the increasingly close relationship of pet owners with their animals [6,7], supporting the need for prudent use of antimicrobial agents. Most of the widely used antimicrobials in veterinary medicine, such as penicillins, quinolones, and cephalosporins [8,9], are categorized as highly or critically important, some of those even as highest priority critically important antimicrobials (HPCIAs) by the World Health Organization [10]. To limit the use of HPCIAs in veterinary medicine, a vast amount of restrictive legislation on antimicrobial use has been passed within the last few years [11,12]. In Germany, an amendment to the Veterinary Home Pharmacy Ordinance (Tierärztliche Hausapothekenverordnung, TÄHAV) came into force in March 2018 [13]. The amendment restricts the use of third- and fourth-generation cephalosporins and fluoroquinolones to cases with a clear medical indication, by mandatorily requesting antimicrobial susceptibility testing. A questionnaire from 2021 found a positive effect of the legislation on veterinarians’ prescribing behaviors [14], with antimicrobial susceptibility testing being performed more often and HPCIAs being used less frequently. Subsequently, an investigation of the prevalence of resistance before and after the amendment was enforced found decreasing trends in resistance profiles of specific bacteria following the amendment [15]. Despite increasing efforts regarding antimicrobial stewardship, antimicrobial treatment prior to breeding has been historically practiced by many breeders and is still common today [16,17,18]. The underlying reason for this is dog breeders’ fear of unsuccessful breeding due to infectious infertility. Indeed, microbiological examinations of the vagina are frequently performed related to canine breeding management [18,19]. The interpretation of bacteriological examination results is challenging, as the canine vaginal flora is a mixed flora [16,19,20,21,22,23,24,25,26,27,28,29] and only *Brucella canis* acts as an obligate pathogen, whereas other bacteria, such as *Escherichia coli (E. coli)*, *Streptococcus canis/* beta-hemolytic streptococci, *Staph. intermedius/pseudintermedius*, and *Pasteurella multocida* [21,30,31,32,33], are considered to be opportunistic pathogens [33,34]. However, these opportunistic bacteria are also frequently isolated from healthy animals [16,21,25,31]. Hence, the sole presence of these bacteria cannot be considered a sign of disease, and bacteriological examinations are of little diagnostic value without corresponding clinical signs of infection [21,35]. Uncertainty about the pathogenicity of bacteria in the individual bitch bears the potential of antimicrobial overuse, especially facilitated by breeders’ pressure on veterinarians due to the fear of subfertility/infertility related to bacterial colonization. The prevalence of antimicrobial resistance in vaginal isolates of dogs is currently unknown. Canine reproduction is of special concern for resistance development, as antimicrobials are often used with questionable indication due to the challenging interpretation of bacteriological examination results. Inappropriate use of antimicrobials in canine breeding was shown to promote the development of multi-resistance and transfer to untreated dogs within the same kennel [36,37]. In addition to public health concerns with unindicated use of antimicrobials, individual health risks must be taken into consideration. Therefore, the aim of this study was to analyze the prevalence of antimicrobial resistance in common vaginal isolates of dogs before and after the TÄHAV amendment.

## 2. Results

A total of 15,155 vaginal swab samples containing 25,626 bacterial isolates were analyzed between 2015 and 2021. The descriptive analysis of the overall dataset is shown in Table 1.

A statistically significant increase in the number of samples (*p* < 0.0001) was observed in TF2. Furthermore, the number of ASTs increased significantly after the TÄHAV amendment (*p* < 0.0001).

From all samples, AST results of 13,373 canine vaginal isolates of *E. coli* (*n* = 5209), beta-hemolytic streptococci (*n* = 4010), and *Staph. intermedius* group (*n* = 4154) were analyzed and included in this study. For TF1, 5042 AST results of the above-mentioned isolates were available, whereas 8279 AST results were available for TF2.

### 2.1. E. coli

**Figure 1:**
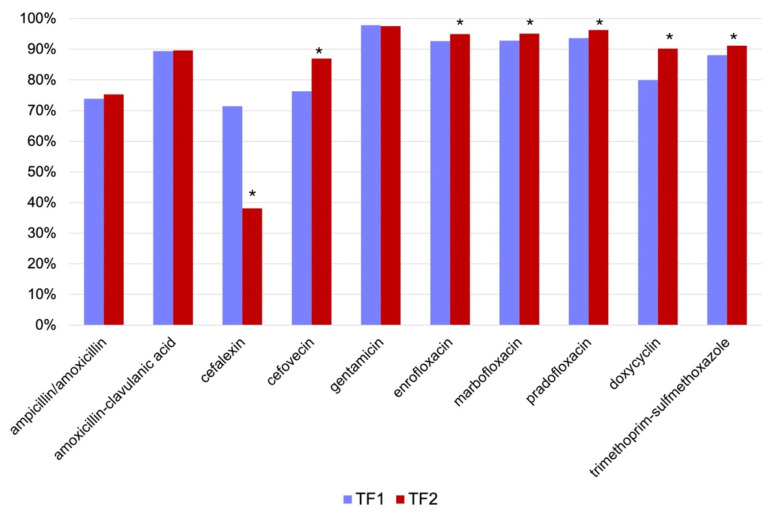
Development of susceptibility of *E. coli* before (time frame 1 (TF1), *n* = 1902) and after (time frame 2 (TF2), *n* = 3307) the 2018 amendment to the Veterinary Home Pharmacy Ordinance (Tierärztliche Hausapothekenverordnung, TÄHAV). Results are presented as percentage (%) of susceptible isolates. Penicillin G was excluded from the analysis due to intrinsic resistance. * *p* < 0.05.

Penicillin G testing was excluded from the statistical analysis, as E. coli shows intrinsic resistance against this antimicrobial. A significant increase in the susceptibility of E. coli was observed for cefovecin, enrofloxacin, marbofloxacin, pradofloxacin, doxycycline, and trimethoprim-sulfamethoxazole. A significant decrease in the number of isolates susceptible to cefalexin was found. No significant changes could be observed for ampicillin/amoxicillin, amoxicillin-clavulanic acid, and gentamicin. Results are shown in Figure 1 and Table 2.

### 2.2. Beta-Hemolytic Streptococci

**Figure 2:**
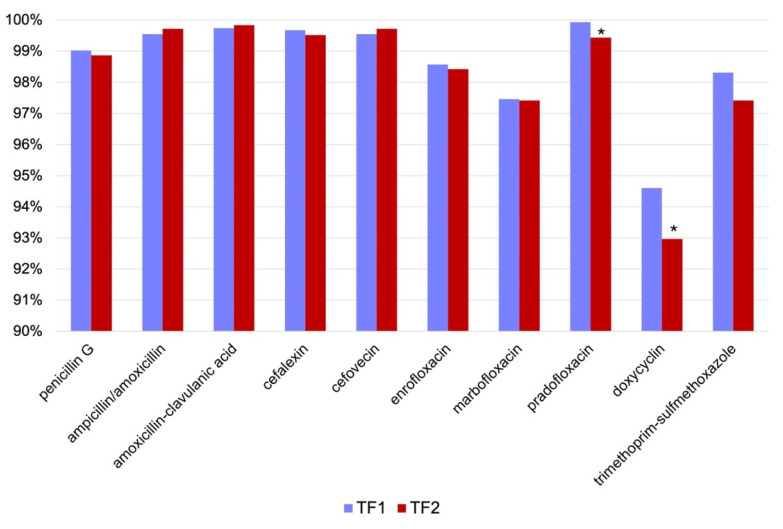
Development of susceptibility of beta-hemolytic streptococci before (time frame 1 (TF1), *n* = 1537) and after (time frame 2 (TF2), *n* = 2473) the 2018 amendment to the Veterinary Home Pharmacy Ordinance (Tierärztliche Hausapothekenverordnung, TÄHAV). Results are presented as percentage (%) of susceptible isolates. Gentamicin was excluded from the analysis due to intrinsic resistance. * *p* < 0.05.

In beta-hemolytic streptococci, antimicrobial susceptibility was generally high, apart from gentamicin, against which beta-hemolytic streptococci are intrinsically resistant [38,39]. Therefore, AST results for gentamicin were excluded from the analysis. Whereas no increase in susceptibility to any of the studied antimicrobials was seen in TF2, a significant decrease in susceptibility to pradofloxacin and doxycycline was observed. Results are shown in Figure 2 and Table 3.

### 2.3. Staph. Intermedius Group

**Figure 3:**
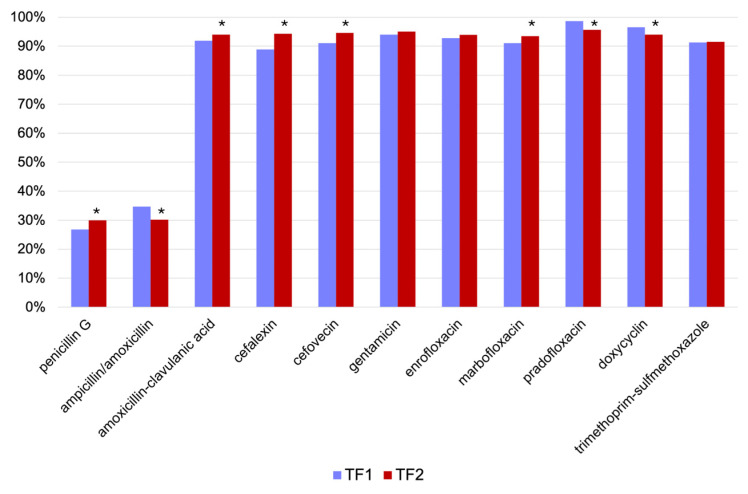
Development of susceptibility of *Staph***.** *intermedius* group before (time frame 1 (TF1), *n* = 1603) and after (time frame 2 (TF2), *n* = 2551) the 2018 amendment to the Veterinary Home Pharmacy Ordinance (Tierärztliche Hausapothekenverordnung, TÄHAV). Results are presented as percentage (%) of susceptible isolates. * *p* < 0.05.

No intrinsic resistance of the *Staph. intermedius* group is described. Susceptibility developed ambivalently in the *Staph. intermedius* group, with a significant increase in the number of isolates susceptible to penicillin G, amoxicillin-clavulanic acid, cefalexin, cefovecin, and marbofloxacin. However, a significant decrease in susceptibility to ampicillin/amoxicillin, pradofloxacin, and doxycycline was found. A slight, however, non-significant increase in susceptibility to gentamicin, enrofloxacin, and trimethoprim-sulfamethoxazole was observed. Results are shown in Figure 3 and Table 4.

## 3. Discussion

This study focused on the effect of the second amendment to the Veterinary Home Pharmacy Ordinance (TÄHAV) in Germany on susceptibility among *E. coli*, beta-hemolytic streptococci, and *Staph. intermedius* group isolates from the canine vagina.

Prudent use of antimicrobials is of particular importance to preserve the efficacy of antimicrobials and prevent the development of resistance [40,41,42]. Moreover, antimicrobials of critical importance for treatment of infectious diseases in humans, issued by the World Health Organization [43], must therefore be restrictively prescribed.

Antimicrobial resistance relies on a multitude of mechanisms, summarized as intrinsic, acquired, and adaptive resistance, with some antimicrobial pathways yet to be investigated. Selective pressure plays a central role in the spread of antimicrobial resistance regardless of the resistance mechanisms. Hence, overuse and misuse of antimicrobials are recognized as the main drivers in the spread of resistance [44,45]. Other factors possibly contributing to resistance, such as complicated adaptive resistance mechanisms in response to certain environmental conditions, signals, or herd resistance mechanisms at the microbial community level, must also be considered [44,45]. Moreover, resistance genes can persist over long periods, even in the absence of selective pressure [45].

Various legislative restrictions and guidelines for prudent antimicrobial use have been introduced in the past few years in European countries (e.g., Belgium, Denmark, Finland, France, Italy) [11]. In Germany, over- and misuse of antimicrobials are theoretically prevented by the TÄHAV amendment, particularly in third- and fourth-generation cephalosporins and fluoroquinolones. However, prudent use might not be strictly complied with when prescribing all HPCIAs, as the amendment has certain weaknesses. Although it stipulates AST for the use of cephalosporins of the third and fourth generation and fluoroquinolones, it does not specify which antimicrobial must be used after AST. The prescribing thereof is legally authorized as long as AST is carried out, regardless of the susceptibility profile of the respective bacteria. It is neither required to await the AST results before the start of the therapy, nor to avoid HPCIAs if the bacteria are susceptible to less critical substances [13].

Vaginal swabs for bacteriological examination are regularly obtained pre-breeding to avoid potential infectious subfertility/infertility [18,19]. Interpreting canine vaginal bacteriological examination results is challenging, as most aerobic bacteria act opportunistically pathogenic [21,24,31,33,34,46], and results do not differ between fertile and infertile dogs. Despite the diagnostic value of sampling in the absence of clinical signs of inflammation being controversially discussed [21,24], prophylactic antimicrobial treatment is still common [16,17,18]. This practice of prophylactic and metaphylactic use is incompatible with prudent use of antimicrobials, especially when considering the global health threat due to antimicrobial resistance. Instead, the necessity of antimicrobial treatment should be thoroughly assessed on a case-by-case basis, based on clinical and gynecological examinations, and avoided if unnecessary.

A clear limitation of the current study is that we did not obtain minimum inhibitory concentrations (MICs) to quantify the isolates’ susceptibility towards antibiotics. Only MICs allow for a quantitative comparison of susceptibility. Moreover, the lack of species- and infection-specific breakpoints in veterinary medicine is a huge concern [47]. Nevertheless, the current study provides for the first time significant insights into the development of the antimicrobial susceptibility of selected bacteria isolated from the canine vagina over time, namely, before and after the TÄHAV amendment. Data analysis was intentionally restricted to the three most important groups of bacteria considered relevant as facultative pathogens in canine reproduction, namely, *E. coli*, beta-hemolytic streptococci, and the *Staph. intermedius* group.

An overall trend towards more susceptible isolates could be observed within this dataset. For *E. coli*, a significant increase in susceptibility to 6 of 10 antimicrobials was observed. A significant decrease in susceptibility was only seen in cefalexin. This is possibly attributable to the introduction of a species-specific breakpoint for canine isolates by the Clinical and Laboratory Standards Institute (CLSI) in 2018 [48], and implemented by the diagnostic laboratory in 2020. Prior to this, a human-derived breakpoint had been used. It seems likely that earlier susceptibility rates (until 2020) did not depict the actual situation in the respective years, but that non-susceptibility to cefalexin was in fact more widespread among *E. coli* isolates than reported. Unfortunately, a verification of this hypothesis is not possible based on the available data.

Beta-hemolytic streptococci generally showed good susceptibility to most antimicrobials. This is in accordance with another study evaluating the effect of the TÄHAV [15]. However, a significant decrease in susceptibility to doxycycline and pradofloxacin was observed. The *Staph. intermedius* group isolates showed variable development in susceptibility, with a significant increase in susceptibility to 5 of 10 antimicrobials and a significant decrease in susceptibility to 3 of 10 antimicrobials. Similar to beta-hemolytic streptococci, a decrease in susceptibility to pradofloxacin and doxycycline was observed. This is of special concern, as pradofloxacin, a third-generation fluoroquinolone for oral application, belongs to the HPCIA group. It was developed for treatment of aerobic and anaerobic infections in small animals and approved in 2011 by the European Union [49,50]. It has enhanced in vitro activity against Gram-positive aerobic bacteria and anaerobic bacteria compared to other fluoroquinolones [51]. In previous studies, MICs against various bacterial pathogens were low [50,51]. However, in another study, single isolates of *E. coli* (5.3%) and *Staph. pseudintermedius* (3.7%) showed higher MICs. Those isolates exhibited either single or double mutations in the quinolone resistance determining regions *gyrA*, *parC*, and *grIA* [52]. The decreased susceptibility to pradofloxacin could be attributable to an increased use of the drug. Unfortunately, however, this cannot be evaluated because no drug sales data for pradofloxacin in Germany are available [53]. Yet, while susceptibility towards pradofloxacin decreased in beta-hemolytic streptococci and the *Staph. intermedius* group, it increased in *E. coli* isolates. The underlying mechanisms are probably more complicated and should be investigated in future research. The decrease in susceptibility to doxycycline in both beta-hemolytic streptococci and the *Staph. intermedius* group isolates might be associated with an increased use of the drug in TF2, possibly because doxycycline was licensed for use in small animals in Germany for the first time in 2017 [54]. Previously, doxycycline preparations licensed for use in humans or other animals had to be prescribed and repurposed for use in dogs. Thus, a possible increase in the use of doxycycline is likely to be attributable to better availability of the drug for use by small animal veterinarians. Moreover, an increased use of doxycycline could also be associated with a change in prescribing patterns among veterinary practitioners due to the restrictive legislation to compensate for the now regulated antimicrobials. However, in Germany, the sale of all listed antimicrobials, including doxycycline, declined from 2015 to 2021, but the official records only capture the total sale of the active ingredients to veterinarians [53]. The antimicrobials prescribed for farm animals probably account for a larger proportion of total sales than the antimicrobials used in companion animals and, specifically, small animals. Hence, an increased use in small animals could be concealed by a decreased on-farm use. Therefore, the sales report is of limited use to estimate the development of prescribing patterns in small animal medicine. It can only be hypothesized that an increase in the use of doxycycline is causative for the decreased susceptibility in TF2.

Another limitation of our study was the lack of clinical history regarding the samples. Microbiological testing is a routine part of canine breeding management, where presented bitches are likely to be healthy dogs. Nevertheless, it cannot be ruled out that these animals were sampled due to a gynecological condition, e.g., vaginal discharge, and/or might have received antimicrobials previously due to an infection. It is not uncommon for veterinarians to submit a sample after treatment with a first-line antimicrobial has failed [55]. The lack of knowledge concerning previous antimicrobial treatment is a limitation that must be considered when interpreting the data. Yet, the database from a commercial laboratory provides a valuable source for resistance monitoring, despite the discussed limitations. The samples were taken by veterinary practitioners nationwide; therefore, these samples are more likely to reflect the actual situation in the field, as opposed to samples taken prospectively in a study setting. The usefulness of large laboratory databases for epidemiological studies was also recently endorsed by other authors [55].

Despite its limitations, our investigation showed a positive effect on antimicrobial susceptibility in the isolates investigated within TF2, most likely attributable to the restrictive legislation. Future studies could evaluate an extended time frame to investigate long-term effects of restrictive legislation and use MICs for a direct, quantitative comparison of antimicrobial resistance. Nevertheless, more specific legislation could be useful to limit the use of HPCIAs strictly to cases with a clear medical indication.

## 4. Materials and Methods

Bacteriological examination results of sent-in samples were provided by a diagnostic laboratory (Laboklin GmbH, Bad Kissingen, Germany). Samples were collected by veterinary practitioners in Germany between 2015 and 2021 and sent to the laboratory for routine diagnostic purposes.

Data provided by the laboratory included date of collection, sample ID-number, species, site of sampling, cultured isolates, bacterial growth grading, and antimicrobial susceptibility test (AST) results. AST results could be canceled by veterinarians, thus were not available for all samples. Moreover, ASTs were not performed for isolates considered apathogenic, e.g., alpha- and non-hemolytic Streptococci. Only samples with the sampling site vagina with an available AST result were included. Unclear labeling, exterior sampling (e.g., vulvovaginal), or multiple sampling sites were reasons for exclusion from the analysis. The number of samples and ASTs were assessed from the overall dataset, whereas analysis of susceptibility was limited to *E. coli*, *Streptococcus canis*/beta-hemolytic streptococci, and *Staph. intermedius* group isolates due to their specific importance as opportunistic pathogens in canine reproduction. The *Staph. intermedius* group summarizes *Staph. intermedius*, *Staph. pseudintermedius*, and *Staph. delphini* because adequate differentiation is only possible using molecular methods [56,57,58].

All data were assigned to two time frames: Time frame 1 (TF1; 38 months duration) from January 2015 to February 2018 (before the TÄHAV amendment) and time frame 2 (TF2; 46 months duration) from March 2018 to December 2021 (after the TÄHAV amendment).

### 4.1. Bacteriological Examination

On the day of arrival at the laboratory, samples were streaked onto non-selective Columbia Agar with 5% sheep blood (Becton Dickinson GmbH, Heidelberg, Germany) and selective media (Endo-Agar, Becton Dickinson GmbH, Heidelberg, Germany) with an inoculation loop using a three-streak pattern. The swabs remained in Soybean-casein digest broth (CASO-Bouillon, Becton Dickinson GmbH, Heidelberg Germany) for enrichment at 36 °C. Isolation from CASO-Bouillon was carried out 16–24 h after incubation on identical agar plates and under the same conditions. Agar plates were incubated at 36 °C under aerobic conditions and checked for growth after 18–24 h. If no signs of growth were visible, the plates were incubated for another 24 h. Colonies of different macroscopic characteristics were subcultured into pure cultures and incubated again for 18–24 h. Subsequently, the final assessment of cultures was performed.

The diagnosis was based on cultural and biochemical parameters.

Further differentiation of isolates was performed by mass spectrometry (matrix-assisted laser desorption/ionization time-of-flight, MALDI-TOF MS, MALDI Biotyper, Bruker Daltonik GmbH, Bremen, Germany).

### 4.2. Antimicrobial Susceptibility Testing

Antimicrobial susceptibility testing was carried out using the broth microdilution method on pure cultures in accordance with the Clinical and Laboratory Standards Institute (CLSI) [48,59,60]. Mueller–Hinton broth and Wilkins-Chalgren medium (Becton Dickinson GmbH/Oxoid GmbH, Wesel, Germany) were used as a nutrient medium. Antimicrobials included in the test panel are listed in the Supplementary Part (Supplementary Table S1). All susceptibility tests were assessed photometrically using Merlin Micronaut MCN 6 (Merlin Diagnostika GmbH, Bornheim-Hersel, Germany). For interpretation of minimal inhibitory concentration (MIC), CLSI breakpoints were used [48,59,60]. The CLSI is currently the only source for veterinary breakpoints [42]. The data obtained included the interpretation of the MIC as either “S” (susceptible), “I” (intermediate), or “R” (resistant); MICs were not provided. As species-specific breakpoints are not available for all bacterial isolates and antimicrobial agents, canine-specific breakpoints were used whenever possible; otherwise, human-derived breakpoints were used [48,59,60].

### 4.3. Descriptive Data Analysis and Statistical Analysis

Microsoft Excel (Microsoft^®^ Excel, Version 16.74, Microsoft Corporation, Redmond, WA, USA) was used for descriptive data analysis and the graphical presentation of data. For data analysis, results were classified as either “susceptible” or “non-susceptible” (intermediate and resistant results). Only antimicrobials of relevance in reproductive medicine (Table 5) were analyzed.

Additionally, Graph Pad Prism 9.0 (Graph Pad Software, Inc., Boston, MA, USA) was used for the statistical analysis. The total number of samples and the number of ASTs within TF1 and TF2 were described and compared. Moreover, due to different durations of both time frames, a mean sample number per month was calculated following testing for normal distribution using the Shapiro–Wilk test. As data for TF1 did not pass the normality test, a Mann–Whitney test was chosen for further comparison of the time frames.

Fisher’s Exact test was used to assess changes in the prevalence of antimicrobial susceptibility, namely, to describe an increase or decrease in antimicrobial susceptibility. The effect size is represented by the Odds Ratio (OR) and 95% Confidence Interval of the OR using the Baptista–Pike Method and the Gart adjusted logit interval. Results were considered as statistically significant at a level of *p* < 0.05.

## Figures and Tables

**Table 1:** Descriptive statistics and comparison of both time frames (TF1 and TF2) with the total number of vaginal swab samples and the respective number of antimicrobial susceptibility tests (ASTs) performed during the investigation periods. Additionally, the median number of samples per month as well as the minimum (min.) and the maximum (max.) number of samples per month analyzed in the respective time frames are presented: TF1 (January 2015 to February 2018, 38 months) and TF2 (March 2018 to December 2021, 46 months). The Mann–Whitney test was used to compare the number of samples and the number of samples with ASTs between both TFs.

	Number of Samples		Number of Samples with ASTs *	
time frame	TF 1	TF 2	TF 1	TF 2
total	5848	9307	5333	8619
median (Q1/Q3)	135.5 (124.8/182.3)	201.0 (178.0/218.0)	126.5 (113.3/168.3)	187.0 (163.0/202.3)
min.–max.	104–255	151–270	96–231	137–247

* AST for at least one of the isolates within a sample; Q1/Q3: 25% percentile/75% percentile.

**Table 2:** Development of susceptibility of *E.coli* isolates before (time frame 1 (TF1)) and after (time frame 2 (TF2)) the 2018 amendment to the Veterinary Home Pharmacy Ordinance (Tierärztliche Hausapothekenverordnung, TÄHAV). Results are presented as total values of susceptible and non-susceptible isolates, and results of the statistical analysis are presented using a Fisher’s Exact text (*p*-value, Odds Ratio (OR), and 95% Confidence Interval (CI)).

Antimicrobial	TF 1		TF 2		*p*	OR	95% CI of OR
	Non-Susceptible	Susceptible	Non-Susceptible	Susceptible			
amoxicillin/ampicillin	498 (26.18%)	1404 (73.82%)	817 (24.71%)	2490 (75.29%)	0.2463	1.081	0.9499–1.230
amoxicillin-clavulanic acid	201 (10.57%)	1701 (89.43%)	342 (10.34%)	2965 (89.66%)	0.8139	1.024	0.8514–1.229
cefalexin	544 (28.6%)	1358 (71.4%)	2047 (61.9%)	1260 (38.1%)	<0.0001	0.2466	0.2185–0.278
cefovecin	451 (23.71%)	1451 (76.29%)	431 (13.03%)	2876 (86.97%)	<0.0001	2.074	1.789–2.398
gentamicin	41 (2.16%)	1861 (97.84%)	80 (2.42%)	3227 (97.58%)	0.5680	0.8887	0.6035–1.295
enrofloxacin	140 (7.36%)	1762 (92.64%)	167 (5.05%)	3140 (94.95%)	0.0008	1.494	1.182–1.880
marbofloxacin	137 (7.2%)	1765 (92.8%)	161 (4.87%)	3146 (95.13%)	0.0006	1.517	1.201–1.921
pradofloxacin	121 (6.36%)	1781 (93.64%)	122 (3.69%)	3185 (96.31%)	<0.0001	1.774	1.365–2.303
doxycycline	382 (20.08%)	1520 (79.92%)	323 (9.77%)	2984 (90.23%)	<0.0001	2.322	1.978–2.730
trimethoprim-sulfamethoxazole	227 (11.93%)	1675 (88.07%)	291(8.8%)	3016 (91.2%)	0.0004	1.405	1.171–1.686

**Table 3:** Development of susceptibility of beta-hemolytic streptococci isolates before (time frame 1 (TF1)) and after (time frame 2 (TF2)) the 2018 amendment to the Veterinary Home Pharmacy Ordinance (Tierärztliche Hausapothekenverordnung, TÄHAV). Results are presented as total values of susceptible and non-susceptible isolates, and results of the statistical analysis are presented using a Fisher’s Exact text (*p*-value, Odds Ratio (OR), and 95% Confidence Interval (CI)).

Antimicrobial	TF 1		TF 2		*p*	OR	95% CI of OR
	Non-Susceptible	Susceptible	Non-Susceptible	Susceptible			
penicillin	15 (0.98%)	1522 (99.02%)	28 (1.13%)	2445 (98.87%)	0.753	0.8606	0.4464–1.617
amoxicillin/ampicillin	7 (0.46%)	1530 (99.54%)	7 (0.28%)	2466 (99.72%)	0.4143	1.612	0.6039–4.3
amoxicillin-clavulanic acid	4 (0.26%)	1533 (99.74%)	4 (0.16%)	2469 (99.84%)	0.4921	1.611	0.4678–5.543
cefalexin	5 (0.33%)	1532 (99.67%)	12 (0.49%)	2461 (99.51%)	0.6186	0.6693	0.2602–1.818
cefovecin	7 (0.46%)	1530 (99.54%)	7 (0.28%)	2466 (99.72%)	0.4143	1.612	0.6039–4.3
enrofloxacin	22 (1.43%)	1515 (98.57%)	39 (1.58%)	2434 (98.42%)	0.7913	0.9063	0.5409–1.516
marbofloxacin	39 (2.54%)	1498 (97.64%)	64 (2.59%)	2409 (97.41%)	>0.9999	0.98	0.6527–1.475
pradofloxacin	1 (0.07%)	1536 (99.93%)	14 (0.57%)	2459 (99.43%)	0.0136	0.1144	0.01075–0.6759
doxycycline	83 (5.4%)	1454 (94.6%)	174 (7.04%)	2299 (92.96%)	0.04	0.7542	0.5767–0.9906
trimethoprim-sulfamethoxazole	26 (1.69%)	1511 (98.31%)	64 (2.59%)	2409 (97.41%)	0.0632	0.6477	0.4068–1.023

**Table 4:** Development of susceptibility of *Staph. intermedius* group isolates before (time frame 1 (TF1)) and after (time frame 2 (TF2)) the 2018 amendment to the Veterinary Home Pharmacy Ordinance (Tierärztliche Hausapothekenverordnung, TÄHAV). Results are presented as total values and percentage of susceptible and non-susceptible isolates, and results of the statistical analysis are presented using a Fisher’s Exact text (*p*-value, Odds Ratio (OR), and 95% Confidence Interval (CI)).

Antimicrobial	TF 1		TF 2		*p*	OR	95% CI of OR
	Non-Susceptible	Susceptible	Non-Susceptible	Susceptible			
penicillin	1173 (73.18%)	430 (26.82%)	1786 (70.01%)	765 (29.99%)	0.0291	1.168	1.016–1.342
amoxicillin/ampicillin	1047 (65.32%)	556 (34.68%)	1781 (69.82%)	770 (30.18%)	0.0026	0.8141	0.7127–0.9300
amoxicillin-clavulanic acid	130 (8.11%)	1473 (91.89%)	154 (6.04%)	2397 (93.96%)	0.0114	1.374	1.080–1.752
cefalexin	179 (11.17%)	1424 (88.83%)	145 (5.68%)	2406 (94.32%)	<0.0001	2.086	1.658–2.619
cefovecin	144 (8.98%)	1459 (91.02%)	139 (5.45%)	2412 (94.55%)	<0.0001	1.713	1.341–2.189
gentamicin	96 (5.99%)	1507 (94.01%)	126 (4.94%)	2425 (95.06%)	0.1564	1.226	0.9374–1.604
enrofloxacin	116 (7.24%)	1487 (92.76%)	155 (6.08%)	2396 (93.92%)	0.1556	1.206	0.9414–1.545
marbofloxacin	143 (8.92%)	1460 (91.08%)	167 (6.55%)	2384 (93.45%)	0.0052	1.398	1.105–1.762
pradofloxacin	22 (1.37%)	1581 (98.63%)	111 (4.35%)	2440 (95.65%)	<0.0001	0.3059	0.1934–0.4823
doxycycline	55 (3.43%)	1548 (96.57%)	153 (6%)	2398 (94%)	0.0002	0.5569	0.4037–0.7622
trimethoprim-sulfamethoxazole	140 (8.73%)	1463 (91.27%)	216 (8.47%)	2335 (91.53%)	0.7761	1.034	0.8264–1.291

**Table 5:** Susceptibility to the following antimicrobials relevant in veterinary reproductive medicine was analyzed.

Antimicrobial Class	Active Ingredients in the Test Panel
beta-lactams	penicillin G
	ampicillin/amoxicillin
	amoxicillin-clavulanic acid
	cephalexin
	cefovecin
aminoglycosides	gentamicin
fluoroquinolones	enrofloxacin
	marbofloxacin
	pradofloxacin
tetracyclines	doxycycline
sulfonamide-trimethoprim combination	trimethoprim/sulfamethoxazole

## Data Availability

The raw data supporting the conclusions of this article are available upon request from Laboklin GmbH & Co. KG due to restrictions (privately owned commercial dataset).

## Supplementary Materials

The following supporting information can be downloaded at https://www.mdpi.com/article/10.3390/antibiotics13100946/s1: Table S1: Full antimicrobial susceptibility test panel.
