# Supplementary material for: How Restrictive Legislation Influences Antimicrobial Susceptibility in Selected Bacterial Isolates from the Canine Vagina
Source: Antibiotics (Basel). 2024 Oct 9;13(10):946. doi: 10.3390/antibiotics13100946 (PMC11504881; doi:10.3390/antibiotics13100946)
Supplement: Supplementary file 1 [file antibiotics-13-00946-s001.zip › antibiotics-3191405-supplementary.pdf]

Table S1: Overview of the full antimicrobial test panel for all dog samples

antimicrobial class	active ingredients in the test panel
**beta-lactams**	penicillin G
	ampicillin
	amoxicillin-clavulanic acid
	cephalexin
	cefoxitin
	cephoperazon
	cefovecin
	cefquinom
**aminoglycosides**	gentamicin
	neomycin/framycetin
	kanamycin
	tobramycin
**macrolides**	spiramycin
	erythromycin
**lincosamides**	clindamycin
	lincomycin
**fluoroquinolones**	difloxacin
	enrofloxacin
	marbofloxacin
	ofloxacin
	pradofloxacin
	orbifloxacin
**amphenicols**	florfenicol
	chloramphenicol
**tetracyclines**	doxycyclin
**sulfonamide-trimethroprim combination**	trimethoprim/sulfamethoxazole
**polypeptide antibiotics**	polymyxin B/colistin
**steroid antibiotics**	fusidic acid
**nitrofurans**	nitrofurantoin
**ansamycins**	rifampicin
